# Role of Hypoxia-Inducible Factor-1α in the Pathophysiology of Non-Alcoholic Fatty Liver Disease Among Obstructive Sleep Apnea Patients: A Case-Control Study

**DOI:** 10.3390/jcm15134861

**Published:** 2026-06-23

**Authors:** Rana Toghan, Tarek A. Salem, Eptehal Dongol, Fatma Rabea A. Hamdan, Omyma Galal Ahmed, Ahlam Mohammed Sabra Ali, Mohammed H. Hassan, Marwa Abdelhady, Rehab H. Abdel-Aziz

**Affiliations:** 1 Medical Physiology Department, Faculty of Medicine, Qena University, Qena 83523, Egypt; rana.ahmed@med.svu.edu.eg (R.T.); fatma.hamdan@med.svu.edu.eg (F.R.A.H.); rehabhemdan@med.svu.edu.eg (R.H.A.-A.); 2 Department of Pathology, College of Medicine, Qassim University, Buraidah 51452, Saudi Arabia; 3 Chest Diseases Department, Faculty of Medicine, Qena University, Qena 83523, Egypt; eptyhal_mohamed@med.svu.edu.eg; 4 Medical Physiology Department, Faculty of Medicine, Assiut University, Assiut 71515, Egypt; omyma@aun.edu.eg; 5 Internal Medicine Department, Faculty of Medicine, Qena University, Qena 83523, Egypt; drahlam.mohamed@med.svu.edu.eg; 6 Department of Medical Biochemistry & Molecular Biology, Faculty of Medicine, Qena University, Qena 83523, Egypt; 7 Department of Internal Medicine, Faculty of Medicine, Luxor University, Luxor 85951, Egypt; dr.marwa21382@gmail.com

**Keywords:** obstructive sleep apnea (OSA), non-alcoholic fatty liver disease (NAFLD), hypoxia-inducible factor 1-alpha (HIF-1α), insulin resistance (IR), obesity

## Abstract

**Background:** Obstructive sleep apnea (OSA) is characterized by chronic intermittent hypoxia (CIH), which causes numerous metabolic changes, leading to non-alcoholic fatty liver disease (NAFLD). Our study explored the suggested role of hypoxia-inducible factor 1-α (HIF-1α) in the pathophysiological mechanisms linking OSA with NAFLD. **Methods:** This case-control study was conducted at the Sleep Disorders Unit at Qena University Hospital from March 2022 to October 2023, including 64 subjects (48 OSA patients; in a secondary analysis, OSA patients were further stratified according to the presence or absence of NAFLD–16 controls) who were subjected to a polysomnography (PSG) for apnea hypopnea index (AHI) and transient elastography for controlled attenuation parameter (CAP) score and liver stiffness measurement (LSM). Serum levels of HIF 1-α, fasting blood glucose, and fasting insulin were measured. **Results:** HIF-1α level showed the highest significant value was in the severe group (*p* = 0.001). Additionally, the severe group had the highest LSM compared to the other groups (*p* = 0.032). OSA patients with NAFLD, compared to OSA patients without NAFLD, showed significantly higher BMI (42.74 vs. 29.11 kg/m^2^, *p* < 0.001), homeostatic model assessment for insulin resistance (HOMA-IR) mean score (3.92 vs. 1.21, *p* < 0.0001), HIF-1α level (6.01 vs. 2.14 ng/L, *p* = 0.045), and the LSM score (5.55 vs. 3.85 kPa, *p* < 0.001). HIF-1α showed significant positive correlations with AHI (r = 0.515, *p* < 0.001), waist circumference WC (r = 0.291, *p* = 0.045), HSI (r = 0.3, *p* = 0.038), and CAP score (r = 0.288, *p* = 0.047). **Conclusions:** Although serum HIF-1α levels were significantly higher in OSA patients with NAFLD and correlated with indices of hepatic steatosis, HIF-1α was not identified as an independent predictor of NAFLD after adjustment for metabolic confounders, suggesting a potential role of hypoxia-responsive pathways in pathophysiology of NAFLD in OSA.

## 1. Introduction

Obstructive sleep apnea (OSA) is a chronic disorder characterized by both chronic intermittent hypoxia (CIH) and sleep fragmentation (SF), resulting in many changes, including dysregulation of glucose metabolism, alterations in lipid metabolism and insulin resistance (IR) [1]. OSA affects approximately 17–34% of adults [2] and its prevalence is about four to five times higher in obese individuals than in the lean subjects [3]. Both OSA and non-alcoholic fatty liver disease (NAFLD) are common metabolism-related disorders. NAFLD is considered a complex metabolic disorder and the most common chronic liver disease among children and adults. Its percentage is around 80% among diabetic and obese individuals [4].

Many studies demonstrate a strong connection between OSA and NAFLD prognosis, suggesting that CIH may be linked to the pathogenesis and severity of the NAFLD [5,6]. Until now, the pathophysiological mechanisms linking both disorders are not clear enough. It is suggested that CIH can cause damage to various target organs by inducing metabolic dysregulation and IR [7], oxidative stress [8], and increasing hypoxia inducible factor 1-α (HIF 1-α) expression [9]. These adaptive mechanisms promote modifications in metabolism to match the oxygen availability [10]. The impact of the CIH effect is evident through abnormal liver enzymes, glucose metabolism, and blood lipids, in addition to observable changes in the pathology of the liver [6].

Connections between OSA and NAFLD lead to complex multifactorial diseases, and the exact role of HIF-1α in the onset and prognosis of NAFLD in OSA patients is still unclear. Our study explores the suggested role of HIF-1α in the pathophysiological mechanisms linking OSA with NAFLD. Also, we aim to clarify if the degree of IR, hepatic steatosis or fibrosis is associated with OSA severity.

## 2. Patients and Methods

This case–control study was conducted at the Sleep Disorders Unit, Chest Disease Department, South Valley University Hospital, from March 2022 to October 2023. This study included 48 cases diagnosed with OSA and not yet treated and 16 controls, ranging from 18 to 60 years old. Among the 48 OSA patients, 34 had NAFLD and 14 did not have NAFLD according to CAP score criteria. The exclusion criteria for both cases and controls were having a known history of vascular events (stroke, unstable angina, coronary artery disease, or acute myocardial infarction), evidence of neoplastic diseases, hepatitis B viral infection, hepatitis C viral infection, cholecystitis, hypothyroidism, Wilson’s disease, or alcoholism. Also, patients with known chronic chest diseases or whose polysomnography study showed central apnea or Cheyne–Stokes disorder were excluded.

Participants were recruited consecutively from eligible patients attending the sleep clinic during the study period. The final sample size was determined by the number of individuals fulfilling the inclusion criteria and consenting to participate. The distribution of participants across study groups reflected the natural prevalence of NAFLD among enrolled OSA patients and was not based on a predefined allocation ratio.

A full medical history, clinical examination, and measurements of the anthropometric parameters were done for all cases and controls. Polysomnography (PSG) was done using SOMNOscreen ™ plus EEG 32, SOMNOmedics America Inc. (Coral Gables, FL, USA). The recorded parameters included the apnea-hypopnea index (AHI), the number of desaturation index (ODI), and the time of desaturations below 90%. The diagnosis of OSA was based on the American Academy of Sleep Medicine (AASM( guidelines criteria as follows: normal (if AHI < 5), mild OSA degree (if 5 ≤ AHI < 15), moderate OSA degree (15 ≤ AHI < 30), and severe OSA degree (if AHI ≥ 30) [11].

Hepatic assessment was done using transient elastography using a FibroScan^®^ device and the hepatic steatosis index (HSI). A controlled attenuation parameter (CAP) score and liver stiffness measurement (LSM) value were obtained using a FibroScan^®^ device (FibroScan, Echosens, Paris, France). LSM value was used to evaluate the presence of fibrosis according to the stages of METAVIR fibrosis, and the cut value is 7.0 kPa [12]. CAP score was expressed in dB/m and its values were used to evaluate the stages of hepatic steatosis as follows: S0: < 238 dB/m, S1: 238–258 dB/m, S2: 259–291 dB/m, and S3: ≥292 dB/m [12].

A venous blood sample (about 6 milliliters) was taken. Later, we obtained the serum by centrifugation at 4000 rpm for 20 min. Measurement of alanine aminotransferase (ALT) and aspartate transaminase (AST) levels was performed and used for the calculation of the hepatic steatosis index (HSI) as (HSI) = 8 × (ALT/AST) + BMI + (2, if diabetes mellitus) + (2, if female).

Fasting blood glucose (FBG) and fasting insulin levels were used to detect the homeostatic model assessment for insulin resistance (HOMA-IR). (HOMA-IR = fasting insulin (microU/L) × fasting glucose (mg/dL)/405). HOMA-IR values above 2.0 show enhanced diagnostic value in discriminating NAFLD from non-NAFLD cases [13].

Serum HIF-1α concentrations were quantified using a commercially available sandwich ELISA kit (purchased from Biospes (Chongqing, China), Cat. No.: BZEK1618, intra-assay CV ˂ 6.9% and inter-assay CV ˂ 8.7%) according to the manufacturer’s instructions. Absorbance was measured using an EMR-500 microplate reader (Labomed, Inc., Los Angeles, CA, USA), and concentrations were calculated from a standard calibration curve.

## 3. Statistical Analysis

Data were analyzed using the Statistical Package for Social Science (SPSS) software program version 25.0 (SPSS Inc., Chicago, IL, USA). Normality testing was carried out via the Shapiro test, and accordingly, parametric or non-parametric tests were used. The data were presented as mean ± SD for the normally distributed data, median with interquartile range (IQR) for the non-normally distributed data. Student’s *t*-test and one-way ANOVA were used for normally distributed variables, whereas Mann–Whitney U and Kruskal–Wallis tests were applied for non-normally distributed variables. The Spearman rho test was used in the correlation analysis. Univariate logistic regression analysis was initially performed to evaluate the association between each clinical and biochemical variable and the presence of NAFLD, and to identify factors independently associated with hepatic steatosis and fibrosis severity using CAP score and liver stiffness measurement (LSM) as dependent variables. Variables showing potential associations were subsequently entered into a multivariate logistic regression model to identify independent predictors. Results were expressed as odds ratios (ORs) or B (the unstandardized regression coefficient) and 95% confidence intervals (CIs). Serum HIF-1α level, BMI, WC, HOMA-IR, age, and sex were included as explanatory variables. Statistical significance was considered at *p* < 0.05.

## 4. Results

This study included 64 subjects (48 cases—16 controls). Their median age is 40.5 (30–44.82) years, and their mean BMI is 38.63 (±10.35) kg/m^2^. There are 42 (66%) males and 22 (34%) females among the total study sample.

Table 1 compares control and OSA patients with different severities. A significant gender difference was observed, with a higher ratio of males in the severe OSA group (100%) (*p* = 0.005). Body mass index (BMI) shows significant statistical differences among moderate vs. severe OSA degrees (34.26 ± 10.64 vs. 44.38 ± 5.01, *p* = 0.026), while WC is between moderate vs. severe OSA degrees (106 ± 25.57 vs. 134.75 ± 8.54) and between moderate vs. control (106 ± 25.57 vs. 112.62 ± 14.99) with (*p* < 0.001).

PSG results show that AHI statistically significantly differs among the four different groups: control, mild, moderate, and severe groups) 3.05 (1.92–4.93), 8.85 (7.72–9.07), 17.4 (15.6–20.02), 47.5 (34.78–56.65), respectively, with (*p* < 0.001)). HOMA-IR does not show significant differences among groups (*p* = 0.539) while FBG level is statistically significantly elevated in the severe OSA group vs. the mild group (114.5 (102.75–150.7) vs. 86.5 (76.5–90.5) mg/dL, *p* = 0.001). Also, the HIF-1α level is statistically significantly different between control vs. severe (5.45 (2.32–12.46) vs. 10.21 (5.6–17.11)), mild vs. severe (1.61 (0.74–3.53) vs. 10.21 (5.6–17.11)), and moderate vs. severe (5.44 (2.25–21.33) vs. 10.21 (5.6–17.11)), (*p* = 0.001).

**Figure 1:**
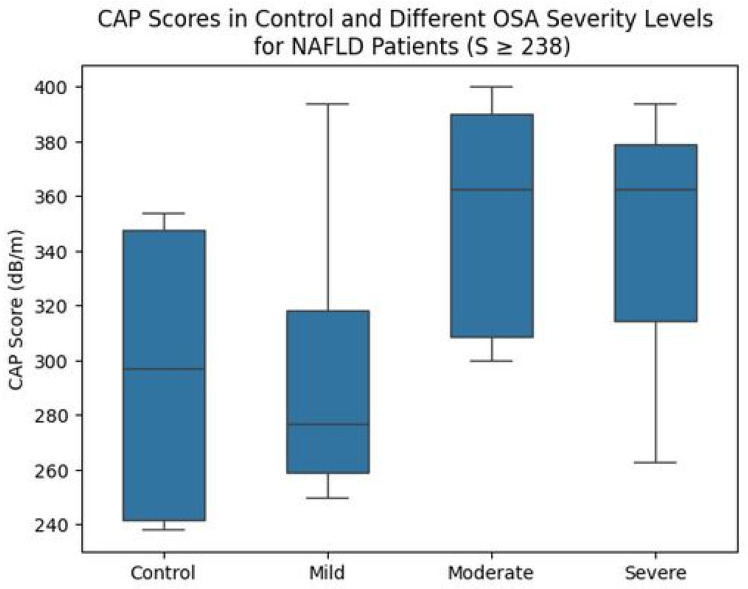
Box plot of the CAP score in control and different OSA degrees who are NAFLD (CAP score ≥ 238). (OSA: obstructive sleep apnea, CAP: controlled attenuation parameter; NAFLD: non-alcoholic fatty liver disease).

**Figure 2:**
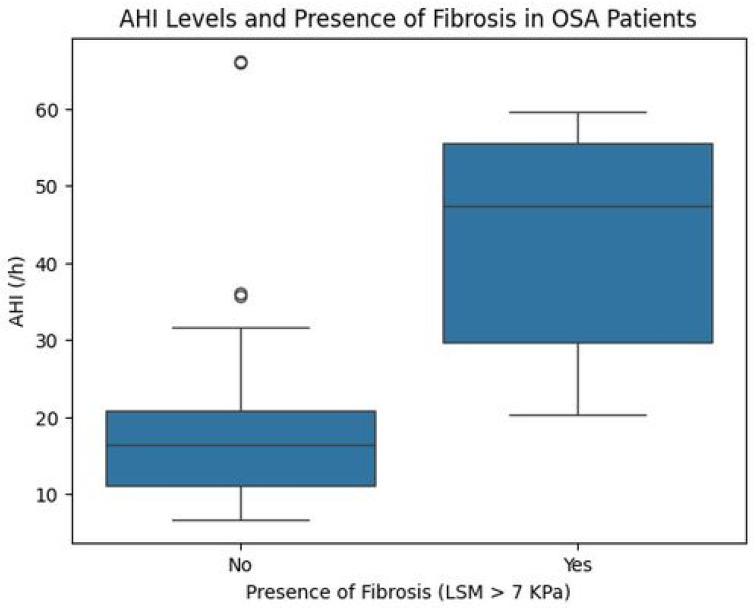
Box blot of the level of AHI in OSA patients with\without liver fibrosis (cut value of LSM 7 kPa) (OSA: obstructive sleep apnea, AHI: apnea-hypopnea index, LSM: liver stiffness measurement).

HSI shows statistically significant differences among the mild vs. moderate (51.02 (±11.6) vs. 45.16 (±13.14)) and mild vs. severe (51.02 (±11.6) vs. 57.46 (±9.36)), with *p* = 0.026. While there is no significant difference in the CAP score when comparing all control and OSA degrees, while comparing the CAP score in control and different OSA degrees who are NAFLD (CAP score ≥ 238), we find a statistically significant difference between control vs. moderate (297 (241.5–347.75 vs. 362.5 (308.5–390.25)) and control vs. severe (297 (241.5–347.75 vs. 362.5 (314.25–379.25)) (*p*= 0.006) as illustrated in Figure 1. The LSM value is significantly different between control vs. severe (4.6 (3.8–5.85) vs. 11 (4.92–15.32)), mild vs. severe (4.9 (4.35–5.6) vs. 11 (4.92–15.32)), moderate vs. severe (5.05 (4.7–5.5) vs. 11 (4.92–15.32)) with (*p* = 0.032). Figure 2 shows the level of AHI in OSA patients with\without liver fibrosis (cut value 7 kPa) (47.5 (29.68–55.55) vs. 16.35 (11.15–20.92) with (*p* < 0.001).

Table 2 illustrates the differences between OSA patients with and without NAFLD (N = 34, 14, respectively). The NAFLD group presented significantly higher BMI (42.74 ± 9 vs. 29.11 ± 8.23 kg/m^2^, *p* < 0.001), NC (44.12 ± 3.94 vs. 40.43 ± 3.99 cm, *p* = 0.005), and WC (131.5 vs. 101.5 cm, *p* < 0.001).

Additionally, the HOMA-IR mean score was a highly statistically significant difference between OSA with and without NAFLD groups (3.92 vs. 1.21, respectively, *p* < 0.0001), HIF-1α level was also statistically significantly elevated in the NAFLD group (6.01 vs. 2.14 ng/L, *p* = 0.045). Also, the LSM score was considerably higher in the NAFLD group compared to OSA patients without NAFLD (5.55 vs. 3.85 kPa, *p* < 0.001).

**Figure 3:**
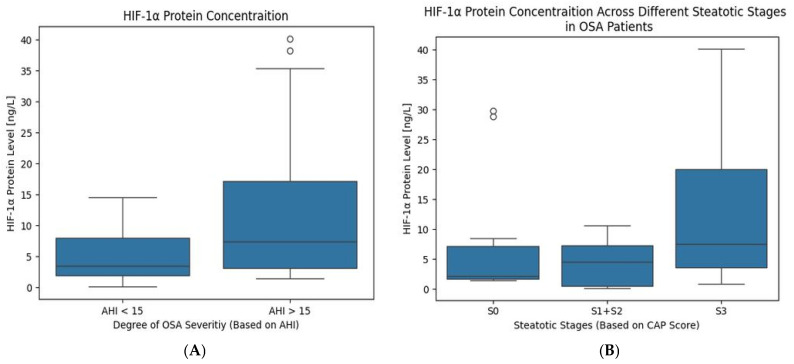
**Levels of HIF 1-α in OSA patients.** They are classified into (**A**) non-severe (if AHI < 15) and severe (if AHI > 15), (**B**) according to steatosis stages (S0 < 238 dB/m, S1: 238–258 dB/m, S2: 259–291 dB/m and S3: ≥292 dB/m) (OSA: obstructive sleep apnea; HIF-1α: hypoxia-inducible factor 1-Alpha; AHI: apnea-hypopnea index).

Figure 3 shows the level of HIF-1α in OSA patients if they are classified into different groups:

(Figure 3A) According to AHI into non-severe (if AHI < 15) vs. severe (if AHI > 15) OSA degrees, (1.61 (0.74–3.53) vs. 7.43 (3.12–17.17) with (*p* < 0.001).

(Figure 3B) According to steatosis stages as S0 (2.14 (1.68–7.13), S1 + 2 (4.56 (0.48–7.24), S3 (7.51 (3.45–19.97)), with (*p* = 0.023).

Table 3 presents the correlation analysis of HIF-1α, HOMA-IR, and CAP scores in OSA patients to other parameters. HIF-1α showed significant positive correlations with AHI (r = 0.515, *p* < 0.001), WC (r = 0.291, *p* = 0.045), HSI (r = 0.3, *p* = 0.038), and CAP score (r = 0.288, *p* = 0.047). Additionally, HIF-1α was correlated with FBG (r = 0.503, *p* < 0.001), AST (r = 0.335, *p* = 0.02) and ALT (r = 0.354, *p* = 0.014).

HOMA-IR was significantly correlated with the HSI (r = 0.521, *p* < 0.001), CAP score (r = 0.626, *p* > 0.001), BMI (r = 0.439, *p* = 0.002), and WC (r = 0.517, *p* > 0.001). CAP score showed a positive significant correlation with BMI (r = 0.53, *p* > 0.001), WC (r = 0.578, *p* > 0.001), and NC (r = 0.355, *p* = 0.013). Also, a significant correlation was detected between CAP score and ALT (r = 0.362, *p* = 0.011) and LSM value (r = 0.464, *p* = 0.001).

Univariate logistic regression analysis demonstrated significant associations between BMI, waist circumference, HOMA-IR, and the presence of NAFLD. After adjustment for potential confounders, waist circumference and HOMA-IR remained significant independent predictors of NAFLD, whereas HIF-1α was not independently associated with disease occurrence as shown in Table 4.

Table 5 identified that in univariate linear regression analysis, BMI, waist circumference, and HOMA-IR were significantly associated with CAP score. After adjustment, HOMA-IR remained as the only significant independent predictor of CAP score. The final model explained 54.3% of the variability in CAP score (R^2^ = 0.543, adjusted R^2^ = 0.477) and was statistically significant overall (F = 8.13, *p* < 0.001). Neither serum HIF-1α levels nor anthropometric parameters showed independent associations with steatosis severity after adjustment for confounding variables.

Table 6 revealed that waist circumference and HOMA-IR were independently associated with liver stiffness values. These findings suggest that central obesity and insulin resistance contribute substantially to liver fibrosis severity. The model explained 50.7% of the variability in LSM (R^2^ = 0.507, adjusted R^2^ = 0.434) and was statistically significant overall (F = 7.02, *p* < 0.001). Serum HIF-1α levels did not demonstrate an independent association with liver stiffness after adjustment for metabolic and demographic factors.

## 5. Discussion

OSA is characterized by CIH, which significantly impacts different metabolic processes [14]. Our study suggests that OSA might be correlated to NAFLD via alternation in HIF-1α, glucose metabolism, and insulin resistance.

One of the adaptive mechanisms of CIH is stabilizing hypoxia-inducible factors (HIFs) and preventing its hydroxylation and ubiquitin-mediated proteasome degradation. This helps in maintaining cellular oxygen homeostasis and ATP levels, but it changes the metabolic process [15].

Some studies found a significant elevation of HIF-1α in OSA patients vs. controls [9,16]; and another study observed that the elevation is higher in moderate to severe OSA cases (AHI ≥ 15) [17]. Our study supports this association, as HIF-1α serum level is positively correlated with AHI, as CIH causes increased expression of HIF-1α in many organs, including pancreatic beta cells. Elevated HIF-1α levels can lead to inflammatory responses and increase reactive oxygen species (ROS) production, which induce IR [18].

Our study supports this association, as FBG is associated with OSA severity. Notably, there is a significant difference in FBG levels between mild and severe OSA degrees, suggesting that OSA severity is independently associated with elevated FBG levels. This difference may be attributed to several underlying mechanisms, including sympathetic nervous system activation or pancreatic inflammation. Sympathetic overactivity promotes hyperglycemia, while pancreatic inflammation may lead to β-cell dysfunction and apoptosis, which may further exacerbate insulin secretion impairment, thus linking OSA to metabolic dysfunction [19].

The relationship between HOMA-IR and OSA severity is unclear and complex. An established correlation between OSA severity and IR—assessed by HOMA-IR—indicates that IR worsens when oxygen saturation decreases [7,19].

On the other side, a study found that whole-body insulin sensitivity does not correlate with the AHI; in contrast, OSA severity is linked to adipose tissue insulin sensitivity, which can produce or worsen IR via changing free fatty acids (FFA) metabolism [20]. This supports that obesity is one of the most effective risk factors for developing HOMA-IR. In our study, there is a positive correlation between HOMA-IR and various obesity-related parameters, including BMI, WC, and NC. However, no significant correlation was found between HOMA-IR and AHI, indicating that while OSA contributes to metabolic disturbances, obesity, especially central obesity, may play a more critical role in developing IR [1]. Additionally, the present study demonstrated that insulin resistance, as reflected by HOMA-IR, was the strongest independent determinant of both NAFLD occurrence and steatosis severity. Moreover, waist circumference emerged as an independent predictor of NAFLD and liver stiffness, highlighting the pivotal role of visceral adiposity in the progression of fatty liver disease. These findings provide evidence that central obesity and insulin resistance remain key drivers of hepatic steatosis and fibrosis.

IR increases the level of FFA in the bloodstream, with subsequent intake by the liver, and also induces de novo lipogenesis (DNL) in the liver due to hyperinsulinemia [21]. In addition, IR stimulates abnormal adipose tissue function, including an abnormality in adipokine secretion, and elevated TNF-α and IL-6 levels. This can highlight the multifaceted interplay between OSA, obesity, and metabolic dysfunction, including IR and explain how the bidirectional relationship between obesity and OSA as a vicious circle increases the prognosis of NAFLD [22,23].

On the other side, one of the changes in metabolic processes resulting from increased HIF-1α expression is enhancing the expression of many genes that involved in glucose uptake and glycolysis, particularly the glucose transporters as GLUT1 and GLUT3. This leads to enhanced hepatocytes uptake of glucose, and glycolysis provides more substrate (pyruvate) for lipogenesis, leading to the accumulation of triglycerides. In addition to promoting DNL, HIFs repress β-oxidation of fatty acids and induce FFA uptake, thus exacerbating the toxic lipid accumulation in the liver and inducing hepatic steatosis [24,25].

Additionally, the level of HIF-1α is further elevated due to local tissue hypoxia resulting from lipid accumulation within adipocytes and hepatocytes, which can reduce local perfusion, establishing a vicious cycle that exacerbates metabolic dysregulation [26]. In our study, we observed a weak but significant correlation between HIF-1α levels at one side and WC, HSI, and CAP score on the other side, highlighting the link between visceral fat accumulation, local tissue hypoxia, and altered hepatic function.

Some studies suggest that hypoxia and HIF-1α play a critical role in the pathogenesis of liver fibrosis via multiple mechanisms, including lipid accumulation and the induction of oxidative stress [25,27]. We noticed a significant difference in HIF-1α level between OSA grades and between NAFLD and non-NAFLD groups in OSA patients. This suggests that HIF-1α is correlated with AHI, indicating the influence of OSA severity on HIF-1α levels. In addition, AHI is associated with the LSM score (an indicator of liver fibrosis), which supports the hypothesis that OSA is implicated in liver fibrosis progression, which increases with the severity of OSA [27]. Absence of difference in AHI between NAFLD and non-NAFLD groups can be explained by the fact that AHI is not an accurate tool to detect the health consequences, while hypoxic burden measures the actual oxygen desaturation, providing a better assessment [28]. Although serum HIF-1α levels were elevated in patients with OSA and NAFLD, supporting the hypothesis that chronic intermittent hypoxia contributes to hepatic steatosis through activation of hypoxia-responsive pathways. However, the observed associations in our study did not remain significant after adjustment for obesity-related and metabolic variables. This suggests that the effect of hypoxia-related pathways on NAFLD may be partially mediated indirectly through metabolic abnormalities triggered by intermittent hypoxia, particularly visceral adiposity and insulin resistance rather than representing an independent pathogenic mechanism, indicating that HIF-1α activation is closely linked to metabolic dysfunction and obesity-associated inflammation.

Finally, other studies found that OSA correlated with increased levels of ALT more than AST as the increase in their levels is approximately 13.3% and 4.4%, respectively [6,29]. In our study, HIF-1α is correlated with both ALT and AST, while the CAP score is associated with ALT enzyme elevation only, which is more specific to hepatocellular injury than AST. Also, ALT and AST are associated with fibrosis. The presence of elevation in hepatic enzymes suggests that NAFLD is progressing to fibrosis [30].

Briefly, CIH, characteristic of OSA, leads to generalized tissue hypoxia. This condition stimulates adaptive mechanisms in tissues, one of which is the increased HIF-1α expression. This increase is correlated with the severity of OSA and NAFLD and is suspected to play a role in the pathophysiological mechanisms underlying HOMA-IR and NAFLD.

**Figure 4:**
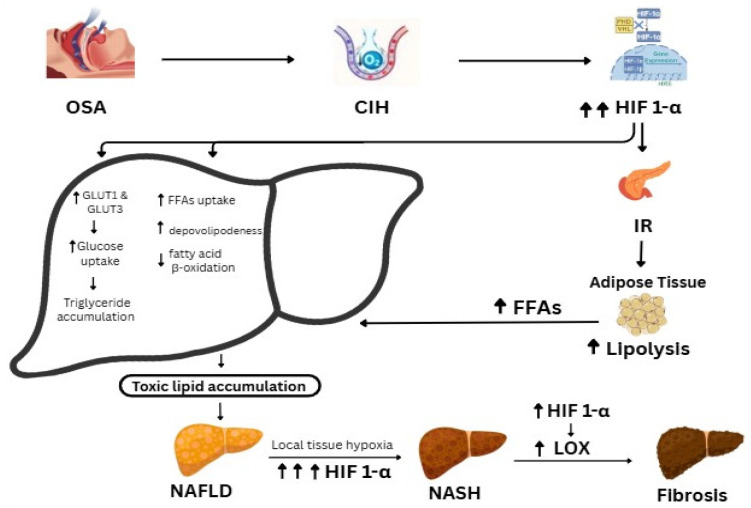
A flow chart illustrates the role of HIF-1α in IR, NAFLD, and NASH in OSA patients (OSA: obstructive sleep apnea; CIH: chronic intermittent hypoxia; HIF-1α: hypoxia-inducible factor 1-Alpha; IR: insulin resistance; FFAs: free fatty acids; NAFLD: non-alcoholic fatty liver disease; LOX: Lysyl Oxidase; NASH: non-alcoholic steatohepatitis.).

The suggested mechanism is that HIF-1α induces IR by altering metabolic processes or stimulating β-cell inflammation. This IR, in turn, promotes lipolysis and increases FFAs. Conversely, in hepatocytes, HIF-1α enhances glucose uptake, FFAs uptake, and DNL, while inhibiting β-oxidation. This results in toxic lipid accumulation within hepatocytes, leading to local tissue hypoxia, which further elevates HIF-1α levels. This cycle stimulates LOX and contributes to NAFLD progression to NASH, as shown in (Figure 4).

All these metabolic changes are suspected to initiate in cases of moderate to severe OSA (AHI > 15), with HOMA-IR and NAFLD preceding the development of metabolic syndrome and its associated features.

## 6. Conclusions

The present study highlights a significant association between circulating HIF-1α levels and both obstructive sleep apnea severity and hepatic steatosis. These findings suggest that hypoxia-responsive pathways may contribute to the metabolic alterations linking chronic intermittent hypoxia to NAFLD. Nevertheless, obesity and insulin resistance emerged as the principal determinants of disease severity, indicating that the contribution of HIF-1α is likely integrated within a broader network of metabolic disturbances rather than acting as an independent driver. Given the cross-sectional nature of the study, further longitudinal and mechanistic investigations are warranted to clarify the causal role of HIF-1α in the development and progression of NAFLD among OSA patients.

### Limitations of the Study

One limitation of the present study is the relatively small sample size, which may have reduced the statistical power to identify an independent association between HIF-1α and NAFLD severity after adjustment for potential confounders In addition, the unequal distribution of participants across study groups may have further limited the ability to detect modest associations, particularly in subgroup analyses and multivariable models. Accordingly, larger, well-powered prospective studies are needed to better delineate the role of HIF-1α in the pathogenesis of NAFLD among patients with obstructive sleep apnea.

Another important limitation is that the control group was predominantly obese, which may have attenuated between-group differences and complicated the isolation of the independent effects of OSA from obesity-related metabolic influences. Moreover, the imbalance between cases and controls reflects the recruitment characteristics of the eligible study population and may have affected the precision and stability of the estimated effects.

For Future Research: Investigating if treatment of OSA with Continuous Positive Airway Pressure (CPAP) would affect HIF-1α level and CAP score. Also, it is recommended to detect if adipose tissue insulin sensitivity is more accurate than HOMA-IR to detect IR in OSA patients.

## Figures and Tables

**Table 1:** Comparison among control and different grades of OSA patents.

		Control (n = 16)	Mild OSA (n = 10)	Moderate OSA (n = 22)	Severe OSA (n = 16)	*p*-Value
Age (years)		39.0 (29.25–43.75)	41.5 (30.25–65.75)	34 (26–50.25)	50.5 (42.25–59)	0.2
Sex	Male	8 (50%)	4 (40%)	14 (63.64%)	16 (100%)	0.005
	Female	8 (50%)	6 (60%)	8 (36.36%)	0	
BMI (Kg/m^2^)		38.23 (±9.48)	39.63 ± 13.72	34.29 ± 10.64	44.38 ± 5.01	0.026 ^$^
NC (cm)		42.62 (±2.09)	42.5 ± 3.41	40.95 ± 3.95	46.25 ± 3.26	<0.001 ^$^
WC (cm)		112.62 ± 14.99	117.5 ±14.32	106 ± 25.57	134.75 ±8.54	<0.001 ^$@^
AHI (/h)		3.05 (1.92–4.93)	8.85 (7.72–9.07)	17.4 (15.6–20.02)	47.5 (34.78–56.65)	<0.001 *^#$^@^
ODI (/h)		3.25 (2.1–7.28)	5.8 (4.72–25.3)	25.95 (7.45–35.33)	45.85 (21.3–59.45)	<0.001 ^#$^@^
SpO2 time < 90% (sec)		0.0 (0.0–0.38)	0.35 (0.1–6.10)	1.05 (0.3–6.65)	15.85 (3.4–42.65)	<0.001 ^#$^^
FBG (mg/dL)		100.5 (97.75–108.0)	86.5 (76.5–90.5)	93.5 (86.5–103.75)	114.5 (102.75–150.7)	0.001 ^#^
Fasting insulin (mU/L)		8.35 (4.45–11.92)	14.15 (8.82–20.88)	8.05 (3.98–18.28)	10.0 (5.68–18.65)	0.363
HOMA-IR		2.05 (1.54–2.98)	3.08 (2.41–3.99)	2.89 (0.82–4.42)	3.04 (1.67–5.66)	0.539
AST (U/L)		20.0 (16.08–28.25)	24.0 (20.5–26.00)	26.0 (20.0–40.0)	23.5 (15.75–54.7.5)	0.424
ALT (U/L)		26.45 (15.38–40.0)	16.5 (15.25–39.5)	26.0 (17.25–53.58)	49.0 (29.5–67.25)	0.204
HSI		50.31 (±12.45)	51.02 (±11.6)	45.16 (±13.14)	57.46 (±9.36)	0.026 *^#^
HIF-1α (ng/L)		5.45 (2.32–12.46)	1.61 (0.74–3.53)	5.44 (2.25–21.33)	10.21 (5.6–17.11)	0.001 ^#$^^
CAP Score (dB\m)		265 (236.25–316.25)	262.5 (251.25–292.75)	300 (191.5–362.75)	350 (295–369.75)	0.184
LSM (kPa)		4.6 (3.8–5.85)	4.9 (4.35–5.6)	5.05 (4.7–5.5)	11 (4.92–15.32)	0.032 ^#$^^

OSA: obstructive sleep apnea, BMI: body mass index, NC: neck circumference, WC: waist circumference, AHI: apnea-hypopnea index, ODI: N. of desaturation index, FBG: fasting blood glucose, HOMA-IR: homeostatic model assessment for insulin resistance, AST: aspartate transaminase, ALT: alanine aminotransferase, HSI: hepatic steatosis index, HIF-1α: hypoxia-inducible factor 1-alpha, CAP: controlled attenuation parameter, LSM: liver stiffness measurement, * mild vs. moderate, ^#^ mild vs. severe, ^$^ moderate vs. severe, ^^^ control vs. severe, ^@^ control vs. moderate.

**Table 2:** Comparison between OSA patients with\without NAFLD.

		OSA with NAFLD (n = 34)	OSA Without NAFLD (n = 14)	*p*-Value
Age		43 (30.25–58.75)	45.5 (27–59.5)	0.946
Sex	Male	24 (70.59%)	10 (71.43%)	0.771
	Female	10 (29.41%)	4 (28.57%)	
BMI (Kg/m^2^)		42.74 ± 9	29.11 ± 8.23	<0.001
NC (cm)		44.12 ± 3.94	40.43 ± 3.99	0.005
WC (cm)		131.5 (116.5–138.5)	101.5 (83.5–114.25)	<0.001
AHI (/h)		20.5 (15.12–35.95)	18.75 (17.23–20.47)	0.901
ODI (/h)		25.2 (7.65–43.15)	26.2 (4.22–34.42)	0.447
SpO2 time < 90% (sec)		1.9 (0.22–10.1)	6.1 (1.12–37.52)	0.241
FBG (mg/dL)		100 (89.5–145.75)	85 (80.25–101.5)	0.008
Fasting insulin (mU/L)		14.15 (8.48–20.88)	5.65 (2.82–7.72)	0.001
HOMA-IR		3.92 (2.59–5.18)	1.21 (0.66–1.66)	<0.001
AST (U/L)		25.5 (17–42.5)	22 (20–34.25)	0.874
ALT (U/L)		39 (16.25–60)	18 (16–44.25)	0.066
HSI		55.91 ± 9.32	37.3 ±9.82	<0.001
HIF-1α (ng/L)		6.01 (3.47–16.31)	2.14 (1.68–7.13)	0.045
CAP Score (dB\m)		359.5 (300–387.75)	182.5 (172.5–201.25)	<0.001
LSM (kPa)		5.55 (5–10.45)	3.85 (2.1–5.15)	<0.001

OSA: obstructive sleep apnea, NAFLD: non-alcoholic fatty liver disease, BMI: body mass index, NC: neck circumference, WC: waist circumference, AHI: apnea-hypopnea index, ODI: N. of desaturation index, FBG: fasting blood glucose, HOMA-IR: homeostatic model assessment for insulin resistance, AST: aspartate transaminase, ALT: alanine aminotransferase, HSI: hepatic steatosis index, HIF-1α: hypoxia-inducible factor 1-alpha, CAP: controlled attenuation parameter, LSM: liver stiffness measurement.

**Table 3:** Correlation between different numerical variables in OSA patients.

	AHI	HIF-1α (ng/L)	HOMA-IR	CAP Score (dB\m) (S)
Variables		Spearman r (*p*-Value)		
BMI (Kg/m^2^)	0.265 (0.069)	0.242 (0.097)	0.439 (0.002) *	0.53 (˂0.001) *
WC (cm)	0.356 (0.013) *	0.291 (0.045) *	0.517 (˂0.001) *	0.578 (˂0.001) *
NC (cm)	0.36 (0.012) *	0.117 (0.229)	0.418 (0.003) *	0.355 (0.013) *
AHI (/hr)	--	0.515 (˂0.001) *	−0.0812 (0.583)	0.119 (0.422)
ODI (/hr)	0.487 (˂0.001) *	0.493 (˂0.001) *	0.238 (0.104)	0.204 (0.164)
SpO2 time < 90% (sec)	0.482(0.001) *	0.167 (0.256)	0.0375 (0.8)	−0.0957 (0.518)
FBG (mg/dL)	0.633 (˂0.001) *	0.503 (˂0.001) *	0.403 (0.004) *	0.456 (0.001) *
Fasting insulin (mU/L)	−0.209 (0.154)	0.117 (0.43)	0.924 (˂0.001)	0.493 (˂0.001) *
HOMA-IR	−0.0812 (0.583)	0.189 (0.199)	--	0.626 (˂0.001) *
AST (U/L)	−0.061 (0.681)	0.335 (0.02) *	0.0845 (0.568)	0.123 (0.404)
ALT (U/L)	0.121 (0.412)	0.354 (0.014) *	0.241(0.099)	0.362 (0.011) *
HSI	0.21 (0.151)	0.3 (0.038) *	0.521 (˂0.001) *	0.661 (˂0.001) *
CAP Score (dB\m)	0.119 (0.422)	0.288 (0.047) *	--	--
LSM (kPa)	0.306 (0.034) *	0.0759 (0.608)	0.179 (0.225)	0.464 (0.001) *

* Significant at *p* < 0.05. AHI: apnea-hypopnea index, ODI: N. of desaturation index, BMI: body mass index, WC: waist circumference, NC: neck circumference, FBG: fasting blood glucose, HOMA-IR: homeostatic model assessment for insulin resistance, AST: aspartate transaminase, ALT: alanine aminotransferase, HSI: hepatic steatosis index, HIF-1α: hypoxia-inducible factor 1-alpha. OSA: obstructive sleep apnea, CAP: controlled attenuation parameter, LSM: liver stiffness measurement.

**Table 4:** Univariate and multivariate logistic regression analysis for predictors of NAFLD among OSA patients.

Variables	Univariate OR (95% CI)	*p*-Value	Multivariate OR (95% CI)	*p*-Value
**HIF-1α**	1.04 (0.97–1.12)	0.231	0.86 (0.72–1.03)	0.104
**BMI**	1.20 (1.07–1.33)	0.001 *	0.85 (0.59–1.23)	0.392
**WC**	1.09 (1.04–1.14)	<0.001 *	1.28 (1.03–1.59)	0.029 *
**HOMA-IR**	2.82 (1.45–5.50)	0.002 *	33.70 (1.55–731.83)	0.025 *
**Age**	1.00 (0.96–1.04)	0.925	0.84 (0.71–1.00)	0.054
**Sex**	0.96 (0.24–3.79)	0.954	0.83 (0.03–25.12)	0.914

* Significant at *p* < 0.05. OR: odds ratio, CI: confidence interval, HIF-1α: hypoxia-inducible factor 1-Alpha, BMI: body mass index, WC: waist circumference, HOMA-IR: homeostatic model assessment for insulin resistance. Multivariable ORs were estimated using Firth penalized logistic regression because the conventional multivariable logistic regression model showed complete separation. The model showed excellent discriminative performance, with an AUC of 1.000, Nagelkerke R^2^ of 0.934, and classification accuracy of 100.0%.

**Table 5:** Univariate and multivariate logistic regression analysis for predictors of CAP score among OSA patients.

Variables	Univariate B (95% CI)	*p*-Value	Multivariate B (95% CI)	*p*-Value
**HIF-1α**	1.24 (−0.88–3.35)	0.246	0.41 (−1.18–2.00)	0.604
**BMI**	4.47 (2.66–6.27)	<0.001 *	2.53 (−0.26–5.33)	0.075
**WC**	2.20 (1.37–3.03)	<0.001 *	0.84 (−0.56–2.23)	0.233
**HOMA-IR**	15.30 (8.78–21.82)	<0.001 *	9.31 (2.58–16.03)	0.008 *
**Age**	0.74 (−0.82–2.30)	0.345	−0.39 (−1.65–0.88)	0.539
**Sex**	1.21(0–51.03–53.46)	0.963	30.81 (−8.95–70.58)	0.125

* Significant at *p* < 0.05. B: represents the unstandardized regression coefficient, CI: confidence interval, HIF-1α: hypoxia-inducible factor 1-Alpha, BMI: body mass index, WC: waist circumference, HOMA-IR: homeostatic model assessment for insulin resistance, CAP: controlled attenuation parameter.

**Table 6:** Univariate and multivariate logistic regression analysis for predictors of LSM among OSA patients.

Variables	Univariate B (95% CI)	*p*-Value	Multivariate B (95% CI)	*p*-Value
**HIF-1α**	−0.00 (−0.11–0.11)	0.939	−0.03 (−0.12–0.05)	0.414
**BMI**	0.08 (−0.04–0.19)	0.179	−0.15 (−0.30–−0.01)	0.420
**WC**	0.09 (0.04–0.14)	<0.001 *	0.14 (0.06–0.21)	<0.001 *
**HOMA-IR**	0.60 (0.23–0.97)	0.002 *	0.38 (0.02–0.74)	0.039 *
**Age**	0.03 (−0.05–0.11)	0.501	−0.01 (−0.07–0.06)	0.857
**Sex**	2.47 (−0.11–5.04)	0.060	3.23 (1.11–5.35)	0.125

* Significant at *p* < 0.05. B: represents the unstandardized regression coefficient, CI: confidence interval, HIF-1α: hypoxia-inducible factor 1-Alpha, BMI: body mass index, WC: waist circumference, HOMA-IR: homeostatic model assessment for insulin resistance, LSM: liver stiffness measurement.

## Data Availability

The data are available from the corresponding author upon reasonable request.

## Abbreviations

OSA: obstructive sleep apnea, CIH: chronic intermittent hypoxia, SF: sleep fragmentation, IR: insulin resistance, NALD: non-alcoholic fatty liver disease, HIF 1-α: hypoxia inducible factor 1-α, PSG: polysomnography, AHI: apnea-hypopnea index, ODI: the number of desaturation index, HIS: hepatic steatosis index, CAP: controlled attenuation parameter, LSM: liver stiffness measurement, ALT: alanine aminotransferase, AST: aspartate transaminase, FBG: fasting blood glucose, HOMA-IR: homeostatic model assessment for insulin resistance.
